# Harvest Time Optimization for Combustion Quality of Different Miscanthus Genotypes across Europe

**DOI:** 10.3389/fpls.2017.00727

**Published:** 2017-05-10

**Authors:** Yasir Iqbal, Andreas Kiesel, Moritz Wagner, Christopher Nunn, Olena Kalinina, Astley F. S. J. Hastings, John C. Clifton-Brown, Iris Lewandowski

**Affiliations:** ^1^Biobased Products and Energy Crops (340b), University of HohenheimStuttgart, Germany; ^2^Institute of Biological, Environmental and Rural Sciences, Aberystwyth UniversityWales, UK; ^3^The School of Biological Sciences, University of AberdeenAberdeen, UK

**Keywords:** miscanthus, harvesting time, genotype, combustion quality, yield loss, nutrient offtake

## Abstract

Delayed harvest can improve the quality of miscanthus biomass for combustion and enhance the long-term sustainability of the crop, despite accompanying yield losses. The aim of this study is to identify the optimal harvesting time, which can deliver improved biomass quality for combustion of novel miscanthus genotypes at various sites across Europe, without high yield losses and without compromising their environmental performance. The relevant field trials were established as part of the European project OPTIMISC with 15 genotypes at six sites across Europe. For this study, the five highest yielding genotypes from each germplasm group and three sites with contrasting climatic conditions (Stuttgart, Germany; Adana, Turkey; and Moscow, Russia) were selected for assessment. The biomass samples were collected between August and March (depending on site) and subjected to mineral and ash content analysis. At Stuttgart, the delay in harvesting time led to a significant variation in combustion quality characteristics, such as N content (0.64–0.21%), ash content (5.15–2.60%), and ash sintering index (1.30–0.20). At Adana, the delay in harvesting time decreased the N content from 0.62 to 0.23%, ash content from 10.63 to 3.84%, and sintering index from 0.54 to 0.07. At Moscow, the impact of delay in harvesting was not significant, except for N, Mg, and ash sintering index. Overall, a delay in harvesting time improved the combustion quality characteristics of each genotype, but at the expense of yield. Yield losses of up to 49% in Stuttgart and Adana and 21% for Moscow were recorded, with variations between genotypes and sites. The harvesting time also affected nutrient offtake, which in turn influences the long-term environmental performance of the crop. The highest N, P, and K offtakes were recorded at Stuttgart for each harvesting time except for final harvest (March), where Moscow had the highest N offtake. This study describes the three criteria (biomass quality, yield losses, nutrient offtake) for determining the ideal harvesting time, which gives the best compromise between dry matter yields and biomass quality characteristics without negatively affecting the environmental performance of the crop.

## Introduction

The challenges of climate change and global warming, linked with the ongoing depletion of fossil fuels, have led researchers and policy makers to search for ways of replacing conventional fuels with renewable and sustainable low-emission fuels. A wide range of biomass resources, such as agricultural and forestry residues, herbaceous dedicated energy crops, woody biomass, and other biodegradable wastes, can be exploited for this purpose (Zabed et al., 2016). The use of lignocellulosic biomass, especially dedicated non-food crops such as miscanthus, switchgrass, and reed canary grass, offers an opportunity to deliver high biomass yields under low-input conditions, potentially also from less suitable agricultural land.

Miscanthus is a very resource-efficient C4 perennial energy grass (Clifton-Brown et al., 2015), which has the potential to grow under marginal conditions (Mi et al., 2014). In Europe, it is the leading perennial energy grass and its biomass is mainly used for combustion to produce heat and electricity. Biomass-based combustion is the preferred utilization option, because it is simple, well known and state-of-the-art technologies are already in place, from small- to large-scale applications (Obernberger and Thek, 2008). By 2020, it is expected that biomass-based energy production will reach 139.5 Mtoe, of which 110.4 Mtoe will be produced in the form of heat and electricity (SWD, 2014). Combustion offers an opportunity to exploit a wide range of biomass resources for this purpose (Arvelakis and Koukios, 2013). However, for an efficient combustion process, biomass quality of specific characteristics is required. The major challenge for the combustion of miscanthus biomass is the low ash melting temperature, which not only reduces the conversion efficiency but also leads to other technical problems such as damage to boiler surfaces (Aho and Silvennoinen, 2004). In addition, it increases the overall operational costs. Therefore, it is important to optimize the constituents of miscanthus biomass for an effective combustion process. For example, high potassium (K), chloride (Cl), and ash contents cause corrosion and fouling (Baxter et al., 2012, 2014; Iqbal and Lewandowski, 2016), and a high moisture content has a direct influence on the heating value (Meehan et al., 2014). For this reason, these biomass constituents need to be kept as low as possible to counter mechanical and technical limitations.

There are several possibilities for enhancing miscanthus biomass quality for combustion. These include technical improvements (Blomberg, 2012), adoption of efficient conversion processes (Wang et al., 2012) and optimization of biomass quality during its production (Iqbal and Lewandowski, 2014). At field level, biomass quality can be improved by adjusting the harvesting time, which can be an efficient and cost-effective measure. Any change in harvest date has a significant influence on both miscanthus biomass composition and yield. However, the response to delayed harvesting varies from genotype to genotype due to differences in phenology (time of flowering and senescence) and morphology (stem thickness, leaf-to-stem ratio). The phenological differences directly influence the nutrient translocation process (Purdy et al., 2015) and the morphological differences affect the leaching of minerals through rainfall (Jørgensen, 1997). An optimal harvest date can improve combustion quality by allowing enough time for the translocation of nutrients back to rhizomes and the leaching of minerals and ash (Iqbal and Lewandowski, 2014). A quality improvement with delayed harvest has been described for the commercially grown standard clone, *Miscanthus* × *giganteus*. However, there is a trade-off between quality improvement and yield, because yield losses of up to 35% can occur between peak yield and a delayed harvest in early spring (Lewandowski and Heinz, 2003). Despite influencing the biomass quality and yield, harvesting time also affects the environmental performance of crop. For example, earlier harvest leads to high nutrient offtake (Smith and Slater, 2011) which subsequently increases the fertilizer input. Many studies have been carried out to evaluate the impact of delaying the harvest time on biomass quality for combustion (Hayes, 2013; Kludze et al., 2013; Bilandzija et al., 2016). However, the mechanisms behind the biomass quality improvement through delayed harvest and the trade-off between quality and yield for different genotypes has not yet been fully described. Therefore, the aim of this study is to identify the optimal harvesting time, which can deliver improved biomass quality of novel miscanthus genotypes at various sites across Europe, without high yield losses and compromising their environmental performance.

For this purpose, three of the six field-trial sites were selected from the European project “OPTIMISC”: Adana (Turkey), Stuttgart (Germany), and Moscow (Russia). From the 15 miscanthus genotypes trialed in this project, three of the highest-yielding were chosen from the germplasm “groups” OPM-3 (*Miscanthus sacchariflorus*), OPM-6 (*M. sacchariflorus* × *Miscanthus sinensis* hybrid), OPM-14 (*M. sinensis*) to be compared with the “standard genotypes” *M.* × *giganteus* (OPM-9) and *M. sinensis* Goliath (OPM-11). The genotypes were harvested at various dates between late summer and early spring. For each harvest date, the quality parameters relevant for combustion (mineral, ash, moisture) were analyzed and the biomass yield assessed.

## Materials and Methods

### Field Trial Description

The field trials were established in 2012 as a part of the EU-funded project OPTIMISC (FP7 No. 289159) with 15 miscanthus genotypes at six sites across Europe. Each genotype was established in a randomized block design with three replications. A full description of the field trials can be found in Lewandowski et al. (2016). From these trials, three sites (Stuttgart, Adana, and Moscow) were selected with the aim of covering a wide range of climatic diversity. From each site, the five most promising genotypes (in terms of dry matter yield) were selected and at least one genotype was also chosen from each species group in order to cover genetic diversity. The genotypes selected are presented in **Table 1**. This study was based on the data from the third growth year.

**Table 1:** Description of miscanthus genotypes used in this study (Lewandowski et al., 2016).

Genotype name	Abbreviation	Provider
*Miscanthus sacchariflorus*	OPM-3	IBERS
*Miscanthus sinensis* × *Miscanthus sacchariflorus* hybrid	OPM-6	IBERS
*Miscanthus* × *giganteus*	OPM-9	IBERS
*Miscanthus sinensis “Goliath”*	OPM-11	IBERS
*Miscanthus sinensis*	OPM-14	WUR

### Site Conditions and Management Practices

The soil texture at Adana and Moscow is silty clay loam to sandy clay loam and at Stuttgart clay loam. **Table 2** shows soil bulk density, stone fraction, and nutrient status [mineral nitrogen (Nmin), phosphorus (P), potassium (K), magnesium (Mg)] at different soil depths for each site.

**Table 2:** Bulk density, stone fraction, and nutrient status for different soil depths for each site.

Site	Depth (cm)	Bulk density (g/cm^3^)	Stone fraction (%)	K_2_O (mg/100g)	P_2_O_5_ (mg/100g)	Mg (mg/100g)	Nmin (mg/kg soil)
Stuttgart	0–30	1.31	6.7	23.5	26.3	26.7	18.0
	30–60	1.66	9.9	8.4	3.6	23.5	3.2
	60–90	1.40	9.9	5.0	3.0	21.7	1.9
Adana	0–30	1.51	10.4	17.7	3.2	15.5	16.6
	30–60	1.64	9.7	12.8	2.0	17.7	14.1
	60–90	1.40	9.7	14.4	3.2	17.7	13.4
Moscow	0–30	1.57	1.8	2.5	11.1	10.8	26.2
	30–60	1.70	3.6	3.6	2.0	16.4	27.0
	60–90	1.40	3.6	3.5	2.3	14.0	na

*na, not available.*

**FIGURE 1:**
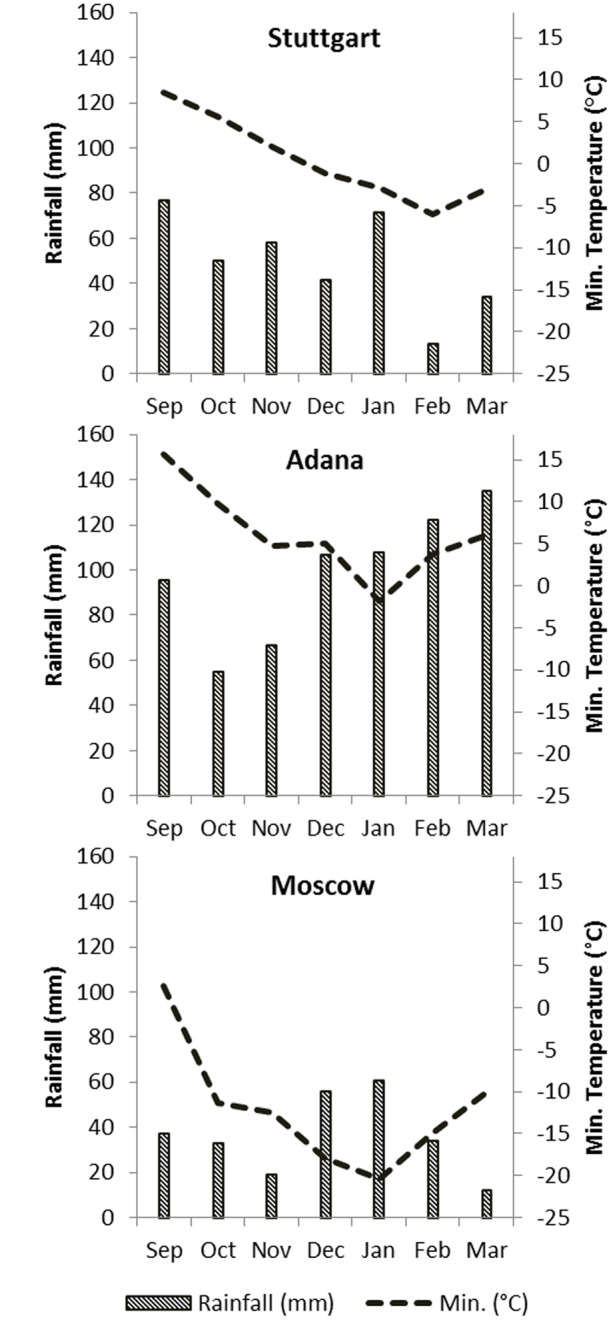
**Monthly rainfall (mm) and minimum air temperature (°C) for each site from September (2014) to March (2015), including irrigation in Adana**.

Meteorological data (monthly rainfall and minimum air temperature from September to March) are shown in **Figure 1**.

Regarding management practices, all the selected sites received the same amount of nutrient application with 60 kg N/ha, 100 kg P/ha, and 140 kg K/ha. Adana also received sufficient irrigation each year to ensure growth was not inhibited of around 200 mm/year.

### Sample Collection

The work was begun in August 2014 with sequential harvests of aboveground biomass, which we called “quality cuts,” starting from August through to January or March, depending on the site. Quality cuts were performed to collect biomass specifically for quality analyses avoiding any damage to the middle 4 m^2^ of each plot, which were used for yield estimations at final harvest. In Stuttgart, quality cuts were performed in August, September, October, November, January, and March. In Adana, they were performed in August, September, October, November, and January. In Moscow, they were performed in August, September, and March because heavy frost killed the aboveground biomass just before the September sampling date and no further quality cuts could be performed until the final harvest in March. Data on morphological characteristics such as leaf-to-stem ratio and stem thickness were collected. The data on leaf-to-stem ratio was collected for every harvesting time, whereas stem thickness was measured only at Stuttgart during final harvest. The same harvesting procedure was adopted at each site. Eight stems were collected randomly from the second-outer row of each plot using manual cutters and leaving stubble of about 5 cm at each harvest date. To ensure the collection was random, a marked pole was used. The quality cut samples were chopped and dried to constant weight (at 60°C for at least 48 h) in a cabinet dryer at each site. The dried biomass samples from Adana and Moscow were shipped to Stuttgart for analysis.

### Chemical Analysis

The milling of all samples was performed in Stuttgart using a SM 200 (Retsch, Haan) cutting mill equipped with a 1-mm sieve and analyzed in the laboratory for nitrogen (N), phosphorus (P), potassium (K), sodium (Na), silicon (Si), calcium (Ca), magnesium (Mg), and ash content. N analysis was carried out by using Vario Macro cube, Elementar Analysensysteme (GmbH, Hanau, Germany) by following the Dumas principle (Naumann and Bassler, 1976/2012; VDLUFA Methods Book III). The extracts were prepared and P, K, Na, Ca, Si, and Mg contents were measured by using ICP-OES (Vista Pro, Varian Inc., Palo Alto, CA, USA). For determination of ash content, samples were kept in Muffle furnace at 550°C for 4 h (Naumann and Bassler, 1976/2012; VDLUFA Methods Book III). The laboratory methods adopted for mineral analysis and ash are described in detail by Iqbal and Lewandowski (2014) and Iqbal and Lewandowski (2016).

The ash-sintering index was developed by correlating biomass composition with ash melting behavior during combustion, based on previous knowledge (Iqbal and Lewandowski, 2016). This index helps to estimate ash melting behavior during the combustion process. An ash-sintering index value (Na + K/Ca + Si) between 0 and 0.20 predicts no to slight sintering risk, between 0.20 and 0.40 slight to strong sintering risk and values above 0.40 strong sintering risk to complete ash melting, depending on biomass composition.

### Statistical Analysis

The laboratory analysis data were used to quantify the impact of genotype, harvesting time, site effect, and the interaction between genotype and harvesting time on combustion-relevant quality parameters. Statistical analysis was performed in SAS version 9.4 (SAS Institute Inc., Cary, NC, USA) using the Proc mixed model with genotype, harvesting time and site effect as fixed and the interaction between genotype and harvesting time as random effects. All variables were tested at a *P*-value of 0.05. The notation of the model is:

(1) $$\begin{array}{c} {\text{y}}_{\text{ijkl}}\text{ = }\mu \text{ + }\alpha_{\text{i}}\text{ + }\beta_{\text{j}}\text{ + }\gamma_{\text{jl}}\text{ + (}\alpha \beta {\text{)}}_{\text{ij}}\text{ + (}\alpha \gamma {\text{)}}_{\text{il}}{\text{ + ei}}_{\text{jkl}} \end{array}$$

where *y_ijkl_* represents the quality parameter for *k*-th replicate of genotype *i*; at site *j* and harvesting time *l*, μ is the general mean of the model; α*_i_* is the effect of genotype *i*; β*_j_* is the effect of site *j*; γ*_jl_* is the effect of harvesting time for site *j* for harvesting time *j*; (αβ)*_ij_* is the interaction between genotype *i* and site *j*; (αγ)*_il_* is the interaction between genotype *i* and harvesting time *l, e_ijkl_* is the error value for corresponding observation.

## Results

### Biomass Composition Analysis Relevant for Combustion Quality

#### N, P, K Content in the Harvested Biomass

The statistical analysis evaluated the impacts of harvesting time, site, genotype, and genotype × harvesting time interaction on biomass composition. The statistical model showed that the impacts of harvesting time, site, and genotype were significant for all biomass quality parameters. The interaction between harvesting time and genotype was also significant for all quality parameters but the interaction between genotype and site was not significant (**Table 3**).

**Table 3:** *P*-values for various quality parameters.

Effect	N	P	K	Ca	Mg	Si	Ash	Sintering index
HT	<0.001	<0.001	<0.001	0.0569	0.0003	<0.001	<0.001	<0.001
Site	<0.001	<0.001	<0.001	<0.001	<0.001	<0.001	<0.001	<0.001
GN	0.006	0.001	0.0006	<0.001	<0.001	<0.001	<0.0001	<0.0001
HT × GN	<0.001	<0.001	<0.001	<0.001	<0.001	<0.001	<0.001	<0.001
Site × GN	ns	ns	ns	ns	ns	ns	ns	ns

*HT, harvesting time; GN, genotype; ns, not significant.*

Overall, with the delay in harvesting time from August to January or March depending on site, the biomass quality characteristics improved significantly as N, P, and K declined with delay.

**FIGURE 2:**
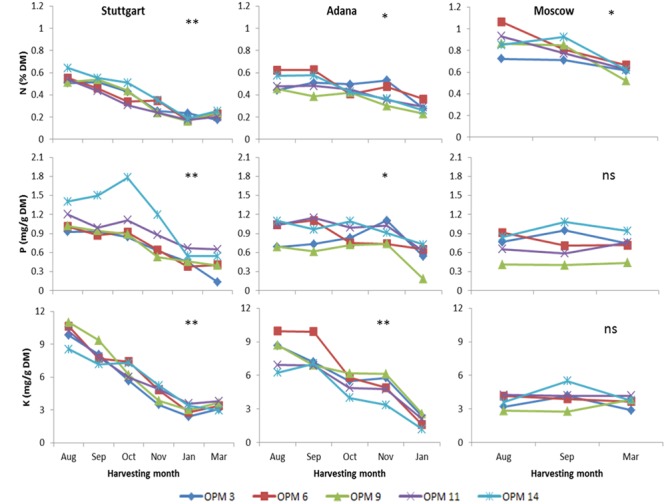
**Nitrogen, phosphorus, and potassium content of biomass from early to late harvest at Stuttgart, Adana, and Moscow for selected genotypes**. Single asterisks (^∗^) indicate a significant impact (*P* = 0.05) of interaction between harvesting time, genotype, and site. Double asterisks (^∗∗^) refer to a highly significant impact and “ns” indicates that the effect was not significant.

For low NO_x_ emissions during the combustion process, it is important to keep the N content in feedstock as low as possible. The N content of all genotypes was significantly higher at Moscow than at the other sites. The response to a delayed harvest also differed depending on site. For example, N content at Stuttgart decreased from 0.64 to 0.21% of dry matter (DM) with delay in harvesting time, which was more rapid than at the other sites. This decrease was significant with the delay until January, but no significant decrease was recorded from January to March. Overall, N, P, and K contents decreased significantly with delay in harvesting time at all sites except Moscow, where a significant decrease was only recorded for N. At final harvest, mean K content of all genotypes was lowest at Moscow (0.11 mg/g DM), followed by Adana (1.95 mg/g DM), and Stuttgart (3.38 mg/g DM). For N, the highest mean content at final harvest (0.61% DM) was recorded for Moscow, followed by Adana (0.28% DM) and Stuttgart (0.22% DM). The highest P content of all genotypes and sites was found in OPM-14 at Stuttgart in October (1.78 mg/g DM) (**Figure 2**).

#### Ash Content and Ash-Forming Elements (Ca, Mg, Si)

**FIGURE 3:**
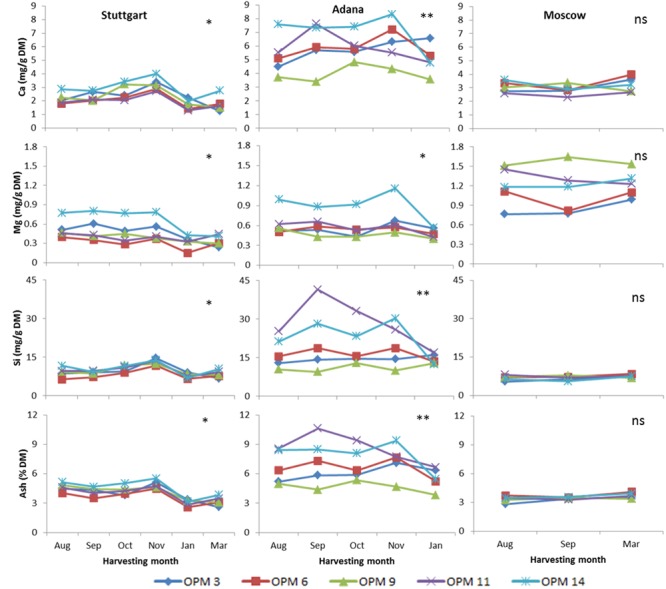
**Ash content and major ash-forming elements for each harvesting time, genotype, and site.** Single asterisks (^∗^) indicate a significant impact (*P* = 0.05) of interaction between harvesting time, genotype, and site. Double asterisks (^∗∗^) refer to a highly significant impact and “ns” indicates that the effect was not significant.

The ash content was highest at Adana, varying from 10.63% for OPM-11 in September to 3.84% for OPM-9 in January. The impact of harvesting time on ash and ash-forming elements (Ca, Mg, Si) was only significant at Stuttgart and Adana. As the major ash-forming element, the Si content followed the same trend as for ash with the delay in harvesting time at each site. No significant difference in ash and Si content between genotypes was recorded at Stuttgart and Moscow, whereas at Adana the variation was significant. At Adana, ash, Si, Ca, and Mg contents were lowest for OPM-9 in comparison to the other genotypes. At Stuttgart, the lowest ash content was recorded in OPM-3 and OPM-6 (**Figure 3**).

### Optimization of Harvesting Time

The ideal harvesting time for combustion depends on the quality of harvested biomass, yield losses, and nutrient offtake. The biomass quality for combustion was evaluated through the development of an ash sintering index based on biomass composition.

#### Ash-Sintering Index

**FIGURE 4:**
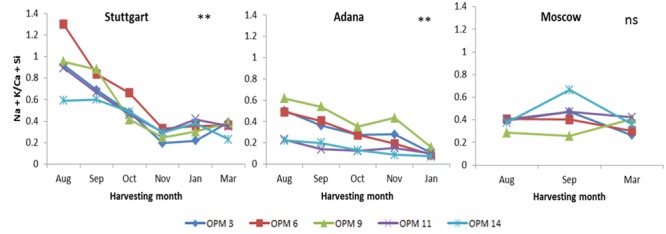
**Values of ash sintering index (Na + K/Ca + Si) for each genotype and harvesting time at Stuttgart, Adana and Moscow.** Double asterisks (^∗∗^) indicate a highly significant impact (*P* = 0.05) of interaction between harvesting time, genotype, and site whereas “ns” indicates that effect was not significant.

Despite high ash content at Adana, the value of the ash-sintering index was below 0.20 at final harvest for all genotypes. This indicates that there will be little to no sintering during combustion when biomass is harvested in January at this site. In some cases, delayed harvest did not have a significant effect on ash sintering. For example, at Adana, no significant improvement in the ash-sintering index was recorded for OPM-11 and OPM-14 with the delay in harvesting time. At Stuttgart, for the January and March harvesting times, the value of the ash-sintering index was below 0.40 for all genotypes (except OPM-11 in January, where ash sintering index = 0.42) (**Figure 4**).

#### Yield Losses

**FIGURE 5:**
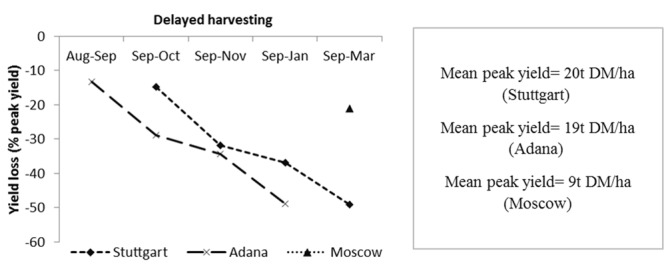
**Yield losses (%) with delay in harvesting time from peak yield for Stuttgart, Adana, and Moscow.** The mean peak yield of all genotypes at each site is also shown.

The yield loss from peak yield was considered one of the criteria for identifying the ideal harvesting time at each site. The peak yield (t/ha) month was taken as the baseline value for the calculation of percentage yield loss with the delay in harvesting time. For Stuttgart and Moscow, September harvest delivered the mean peak yield of all genotypes whereas for Adana, August was considered as peak yield month. At Stuttgart, the yield losses were below 40% when harvesting was delayed from September to January (**Figure 5**), whereas at Adana they reached 49% from peak yield (August) to final harvest in January. For Moscow, the yield loss from the peak-yield month (September) to final harvest (March) was 21%. As there were no yield data between September and March, Moscow is not presented in (**Figure 5**).

#### Nutrient Offtake (N, P, K)

Another important factor for the identification of optimal harvesting time is nutrient offtake, because it directly influences the long-term environmental performance of the crop. High nutrient offtake at a specific harvesting time not only effects biomass quality but also leads to high fertilizer inputs. After the peak-yield month, nutrient offtake significantly decreased at each site with the delay in harvesting time. The highest N, P, and K offtakes were recorded at Stuttgart for each harvesting time, except for the final harvest (March) where Moscow had the highest N offtake (**Table 4**). In Stuttgart and Moscow, the highest N (101.2 and 72.9 kg/ha, respectively) and P offtakes (21.3 and 6.7 kg/ha, respectively) were recorded in September, whereas in Adana the highest offtakes were in August (N = 83.9 kg/ha, P = 15 kg/ha) (**Table 4**). The highest K offtake was recorded in August in Stuttgart (168.3 kg/ha) and Adana (131.4 kg/ha), but in September in Moscow (36.3 kg/ha) (**Table 4**). The high N, P, and K offtakes at Stuttgart can be explained by the high initial nutrient loading at this site.

**Table 4:** Mean nutrient offtake (N, P, K) at each site and harvesting month for all five genotypes.

Harvesting month	N (kg/ha)			P (kg/ha)			K (kg/ha)		
	Stuttgart	Adana	Moscow	Stuttgart	Adana	Moscow	Stuttgart	Adana	Moscow
August	91.4	83.9	39.0	18.3	15.0	3.2	168.3	131.4	15.6
September	101.2	71.9	72.9	21.3	13.0	6.7	162.0	107.3	36.3
October	77.2	45.1	na	20.3	9.3	na	124.8	54.0	na
January	49.0	27.8	na	12.3	5.2	na	74.8	19.1	na
March	27.9	na	42.5	5.2	na	4.9	43.9	na	25.7

*na, not available.*

## Discussion

**FIGURE 6:**
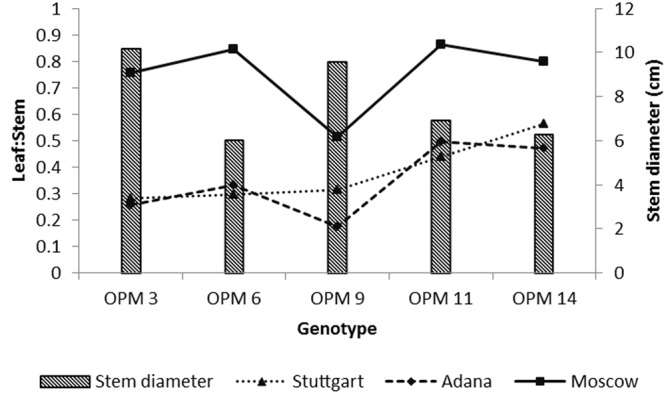
**Leaf-to-stem ratio (shown as lines) for selected miscanthus genotypes at three sites (mean of all harvest dates) and stem diameter (shown as bars) for Stuttgart only (final harvest)**.

This study assessed the variation in biomass quality characteristics between genotypes, mainly due to morphological and phenological differences such as stem diameter and time of flowering and senescence. For the morphological differences, stem thickness and leaf-to-stem ratio play a key role in determining biomass quality for combustion at a specific harvesting time. The genotypes assessed can be listed in the following order of stem thickness OPM-3 > OPM-9 > OPM-11 > OPM-14 > OPM-6. The thick-stemmed genotypes (*M. sacchariflorus* and *M.* × *giganteus*) showed a low leaf proportion (**Figure 6**).

The leaves have high mineral and ash contents, therefore a low leaf proportion is favorable for combustion (Baxter et al., 2014). The low leaf proportion of *M.* × *giganteus* may explain why this genotype had the lowest ash content at final harvest in Adana and Moscow. At Stuttgart, it was only slightly higher than the genotype with the lowest ash content. Stem diameter is an important morphological characteristic because it directly influences the rate of leaching. Other studies have found that thin-stemmed genotypes (*M. sinensis*) show more efficient leaching of minerals than thick-stemmed genotypes (*M.* × *giganteus*) (Jørgensen, 1997; Iqbal and Lewandowski, 2014). In our study, the thin-stemmed OPM-6 showed a rapid improvement in sintering index with delayed harvesting. It is assumed that it is the leaching of K which leads to this improved sintering index, because the same trend was found for K as for the sintering index with delay in harvesting time. From the literature, it is also evident that the rate of fouling and sintering during combustion is determined by the K content (Blomberg, 2007). In plants, K is present as a soluble ion (Jørgensen, 1997). Therefore the K content of biomass is largely influenced by stem thickness and rate of leaching. Kludze et al. (2013) recorded up to 31% reduction in K content of miscanthus through translocation and efficient leaching with winter precipitation with delayed harvest. Based on our own results, we conclude that genotypes with low leaf share and thin stems would be most suitable for combustion purposes. However, for the genotypes tested here, low leaf share seems to be accompanied by thick stems. For example, *M.* × *giganteus* had low leaf to stem ratio but also develop thick stems. Therefore, future breeding program should focus on developing combustion specific genotypes with thin stems and low leaf share.

The variation in leaf-to-stem ratio between genotypes at the three sites shows that, in addition to genotypic variation, there is also a site affect. This site effect can be explained through differences in rainfall, because the timing and amount of rainfall affects the leaching rate of minerals (Iqbal and Lewandowski, 2014). For example, Adana had highest rainfall from October to January, which are the months most relevant for leaching because the biomass is senescent during this period. This led to a rapid improvement in sintering index before final harvest. The significantly highest ash content of all genotypes at Adana compared to the other sites is another indication of the site effect. This can be explained by differences in soil type. The soluble silica (Si) content varies with soil type. For example, clay soils have high soluble Si content, which subsequently leads to high uptake of Si (Bakker and Elbersen, 2005). The high soluble Si content in soil at Adana may have caused the comparatively high ash content of the harvested biomass.

Phenology is another important factor that explains the genotypic variation between sites. In this study, the *M. sinensis* types flowered earliest, followed by the hybrids and then *M.* × *giganteus*. Genotypes with early flowering and senescence, such as *M. sinensis*, delivered higher biomass qualities than genotypes with late flowering and senescence, such as *M.* × *giganteus*, because the time of flowering directly influences the nutrient relocation process. Early flowering genotypes initiate the relocation of nutrients earlier and complete it before winter frost kills the stems (Jensen et al., 2016). The time of flowering and senescence for these genotypes is described by Nunn et al., unpublished.

In the results section, three criteria (biomass quality, yield losses, nutrient offtake) were described for determining ideal harvesting time, which gives the best compromise between dry matter yields and biomass quality characteristics without negatively affecting the environmental performance of the crop. However, no hard criteria in terms of biomass quality, yield losses or nutrient offtake can be set for the identification of the ideal harvesting time, because threshold values have not yet been fully defined for miscanthus. From a biomass quality perspective, the N content of most of the genotypes at final harvest at Stuttgart and Adana falls within the defined threshold limits of the European pellet norms (ENplus A1). Therefore, N content could be used as one biomass quality parameter for identifying the ideal harvesting time. Delayed harvesting can lead to biomass yield losses and also reduce net energy yield ha-1. Early harvesting (November) of miscanthus biomass will lead to a higher net energy yield (GJ/ha) for combustion than delayed harvesting, as reported by Kiesel et al. (2017). However, it not only influences the thermal conversion of biomass but also compromises the nutrient balance of the crop by increasing nutrient offtake. In this study, nutrient offtake (N, P, K) was reduced by up to 70% at Stuttgart through delay in harvesting from August to March. In addition, it is practically not possible to fully close the nutrient cycle in the combustion chain, as can be done in the biogas chain through the recycling of biogas digestates as fertilizer. During combustion, N is lost and currently ash is not allowed as fertilizer as it is classified as waste. Results of this study indicate that delayed harvesting is accompanied by lower nutrient offtakes. In terms of nutrient use efficiency, it is preferable to harvest the biomass as late as possible.

Based on the results of this study, January harvesting can be recommended at Stuttgart for all genotypes, because there was no significant improvement in quality characteristics, especially N and K content, from January to March. However, a delay in harvest from January to March led to additional yield losses of about 12% and the nutrient offtake in January was already reduced by up to 46% compared to August. For some genotypes, such as OPM-3, OPM-9, and OPM-14, even a harvest in November is thinkable, since the N content and sintering index was low at this harvest dates and further improvements by delaying harvest might not be justified by the additional yield losses. However, earlier harvest will lead to higher moisture content (Kiesel et al., 2017) and requires adapted harvest procedure (e.g., windrowing and wilting on field). Biomass with high moisture content poses some additional challenges, such as increased logistics costs and a high risk of spoilage and self-heating during storage. In addition, in case of early harvest, nutrient offtake was also high which compromised the environmental performance of crop. For Adana, January (which was final harvest) can also be recommended for all genotypes, because the biomass quality not only met the threshold values for N content set by European pellet norms (ENplus A1), but also the sintering index was below 0.2. At this harvesting time, the N offtake was reduced by 67% compared to early harvest (August), but yield losses were comparatively high (49%). The results for Adana show that genotypes other than those investigated here would probably be more suitable for biomass production for combustion. Genotypes better adapted to drought conditions could make better use of the biomass production potential.

In Moscow, where no harvesting was possible between September and March due to heavy snowfall, the absence of significant compositional changes through leaf fall or relocation of nutrients can be explained by the short vegetative period. The harsh frost and the mean temperature well below 0 (-12°C) for most of this period (September to March) led to no further crop development and compositional changes. Purdy et al. (2015) found that long periods with temperatures below 0 kill the aboveground stems and negatively affect remobilization of nutrients back to rhizomes. Therefore, there was no significant biomass quality improvement through a delay in harvesting time. However, delaying the harvest until March improved the environmental performance of the crop by reducing N offtake up to 42% (compared to September) with yield losses of only 21%. This indicates that March could be more appropriate than an early harvest, but still the N content is very high compared to the other sites. From the results of this study, we conclude that genotypes which are adapted to short vegetation period and are early senescing need to be developed for sites like Moscow. This would allow the crop to complete the growth cycle more and actively relocate nutrients before first harsh frosts occur. This would help to improve the biomass quality for combustion.

At each location, the yield loss through delayed harvest is mainly due to leaf fall, stem breakage, and inefficient harvesting and collection especially caused by broken stems lying on the ground. However, no specific data on leaf fall and stem breakage were collected from early to late harvest.

From the above discussion, it can be concluded that harvesting time should be decided on and adjusted according to the prevailing weather conditions and thus may vary from one region to another. For example, in many miscanthus-growing regions in the northern hemisphere, frequent rainfall in March, in combination with the thawing effect, may cause soil softening and make the use of harvesting machinery difficult. Under such conditions, minor improvements in combustion quality at the expense of surface damage and soil compaction will not be worthwhile. Therefore, in such a scenario, an early harvest can be performed before the start of the wet season to avoid any soil damage.

## Author Contributions

YI was leading the writing process. MW, AH, JC-B, CN, and OK provided valuable input for improving the manuscript. Furthermore, AK provided support in discussion of the results. IL added valuable contribution to each chapter and in manifold discussions.

## Conflict of Interest Statement

The authors declare that the research was conducted in the absence of any commercial or financial relationships that could be construed as a potential conflict of interest.
